# SGLT2 Inhibitors in Hypertrophic Cardiomyopathy: Emerging Evidence and Putative Mechanisms

**DOI:** 10.3390/biom16060873

**Published:** 2026-06-15

**Authors:** Khrystyna Ryabenko, Valérie Schini-Kerth, Patrick Ohlmann, Elena Galli

**Affiliations:** 1 Cardiology Unit, Cardiac Thoracic and Vascular Department, IRCCS Azienda Ospedaliera-Universitaria di Bologna, 40138 Bologna, Italy; 2 Department of Medical and Surgical Sciences (DIMEC), Alma Mater Studiorum, University of Bologna, 40126 Bologna, Italy; 3 UR 3074—Translational CardioVascular Medicine, Faculty of Pharmacy, University of Strasbourg, 67000 Strasbourg, France; valerie.schini-kerth@unistra.fr (V.S.-K.);; 4 Division of Cardiovascular Medicine, Nouvel Hospital Civil, Strasbourg University Hospital, 67000 Strasbourg, France; 5 UMR 7357—ICube, CNRS—University of Strabourg, 67084 Strasbourg, France

**Keywords:** gliflozines, hypertrophic cardiomyopathy, inflammation, reactive oxygen species, sodium–glucose cotransporter 2 inhibitors

## Abstract

Hypertrophic cardiomyopathy (HCM) is the most common inherited myocardial disorder and a major cause of heart failure (HF) and sudden cardiac death. Although sarcomeric gene mutations initiate the disease, increasing evidence identifies oxidative stress, mitochondrial dysfunction, and maladaptive nutrient signaling as key drivers of disease progression. Enhanced reactive oxygen species (ROS) production in HCM promotes energetic impairment, calcium mishandling, fibrosis, and the activation of pro-hypertrophic pathways, while disrupting protein quality control and endothelial function. Despite recent therapeutic advances, effective disease-modifying strategies targeting these molecular mechanisms remain limited. Sodium–glucose cotransporter 2 inhibitors (SGLT2i), originally developed for type 2 diabetes, have demonstrated robust cardioprotective effects in HF independent of glycemic control. Beyond their renal actions, SGLT2i modulate myocardial metabolism, reduce oxidative stress, improve mitochondrial function, restore sodium and calcium homeostasis, and attenuate inflammation and maladaptive mTOR activation. Emerging preclinical and translational data suggest that these pleiotropic mechanisms may counteract key pathophysiological processes underlying HCM. This review summarizes the molecular interplay between oxidative stress and hypertrophic remodeling in HCM and explores the rationale for SGLT2 inhibition as a potential disease-modifying therapeutic strategy.

## 1. Introduction

Hypertrophic cardiomyopathy (HCM) is the most common inherited myocardial disorder and a major cause of heart failure (HF) and sudden cardiac death [1]. Nearly 60% of patients with HCM have autosomal dominant disease, caused by mutations in cardiac sarcomeric proteins such as the beta-myosin heavy chain (MYH7) and myosin-binding protein C (MYBPC3), and to a lesser extent in troponin codifying genes such as cardiac troponin I and T (TNNI3, TNNT2), the tropomyosin alpha-1 chain (TPM1) and myosin light chain 3 (MYL3). In 5 to 10% of cases, the hypertrophic phenotype is due to phenocopies such as inborn error of metabolism, neuromuscular disease, mitochondrial disease, malformation syndromes, and amyloidosis, whereas 25–30% of HCM are of unknown origin [2]. Although pathogenic variants in sarcomeric proteins initiate the disease, genotype alone does not fully explain the heterogeneity in clinical expression, progression and adverse outcomes [2]. Increasing evidence suggests that secondary mechanisms including oxidative stress, mitochondrial dysfunction, inflammation, and dysregulated nutrient signaling act as critical amplifiers of the primary genetic defects.

The hypercontractile phenotype characteristic of HCM imposes a sustained energetic burden on cardiomyocytes, promoting mitochondrial reactive oxygen species (ROS) production and redox imbalance [3]. Oxidative stress, in turn, disrupts calcium homeostasis, impairs protein quality control [4], activates pro-hypertrophic signaling pathways such as mammalian target of rapamycin (mTOR) and mitogen-activated protein kinase (MAPK), and contributes to fibrosis and diastolic dysfunction [5]. Thus, oxidative stress may represent a central pathophysiological hub linking sarcomeric mutations to maladaptive remodeling.

Sodium–glucose cotransporter 2 inhibitors (SGLT2i), initially developed for type 2 diabetes, have demonstrated robust cardiovascular benefits in HF, independent of glucose lowering effects [6,7]. Beyond their renal effects, SGLT2i modulate nutrient-deprivation signaling, mitochondrial function, sodium and calcium handling, inflammation and redox homeostasis [8,9]. These pleiotropic properties raise the possibility that SGLT2 inhibition could interrupt the oxidative–metabolic amplification loop that drives disease progression in HCM.

This review examines the molecular interplay between oxidative stress and hypertrophic remodeling in HCM and discusses the mechanistic rationale for SGLT2 inhibitors as potential disease-modifying agents.

## 2. Oxidative Stress in Hypertrophic Cardiomyopathy

Reactive oxygen species, such as the superoxide anion radical (O_2_•-), hydrogen peroxide (H_2_O_2_), and the hydroxyl radical (OH•), are signaling molecules that mediate post-translational modifications with pronounced effects on multiple cellular function. In HCM, genetic mutations induce changes in sarcomeric protein structure and function. The increased myofilament Ca^2+^ sensitivity elevates adenosine triphosphate (ATP) hydrolysis for a given cytosolic Ca^2+^ load and increases reduced nicotinamide adenine dinucleotide (NADH) oxidation, without proportional Ca^2+^-driven stimulation of dehydrogenases in the Krebs cycle. This bioenergetic mismatch raises adenosine diphosphate (ADP) and results in the accumulation of H_2_O_2_ [10], which has been identified as the primary consequence of HCM and plays a causal role in the initiation of cardiac dysfunction [11]. Increased oxidative stress markers have been found in several animal models of HCM, including mutations encoding β-MYH7 [12], MYBPC3 [13], TNNT2 [14] and TPM1 [15]. Despite animal studies (in vivo and in vitro) relying on homozygous mutations that do not perfectly mirror most heterozygous mutations observed in clinical practice, oxidative stress markers have also been found in the heart and serum of HCM patients. In the myocardial biopsies of patients with HCM, Lynch et al. found significant redox imbalance, as shown by the increased oxidized-to-reduced glutathione ratio and higher lipid peroxidation markers [16]. Nuclear hypertrophy and oxidative stress damage of DNA have also been observed in endomyocardial biopsies of patients with HCM compared to healthy controls [17]. A recent integrated omics analysis of RNA-sequencing datasets from the Gene Expression Omnibus (GEO; https://www.ncbi.nlm.nih.gov/geo/, accessed on 19 February 2026) have revealed the overexpression of oxidative stress-related genes in HCM compared with healthy controls, along with their associations with key hub genes. By modulating the immune microenvironment, altering myocyte metabolism, disrupting mitochondrial integrity, reducing oxidative phosphorylation, and activating signaling cascades that promote hypertrophy and fibrosis, ROS are drivers of pathological remodeling in HCM [18,19].

### 2.1. ROS-Induced Alteration in Energy Metabolism

In HCM, elevated ROS and the significant reduction in anti-oxidants such as superoxide dismutase, catalase, glutathione reductase and glutathione peroxidase, expose mitochondria to increased oxidative stress [20]. ROS have also been shown to interact with mitochondrial DNA, proteins and lipids, inactivating the electron transport chain complex (ETC) and contributing to the impairment of mitochondrial respiration [21]. Through integrated omics analysis of myocardial samples, Ranjbarvaziri et al. revelated that HCM was associated with major metabolic derangements, including impaired fatty acid oxidation, reduced levels of glucose and glycolytic metabolisms [22]. These alterations are related to the downregulation of genes involved in mitochondrial energy metabolism, such as ETC subunits, and overall reduced phosphocreatine (PCr)/ATP ratios. Interestingly, [^11^C] positron emission tomography analysis showed that the PCr/ATP ratio, a marker of lower cardiac efficiency, was significantly reduced in both symptomatic and asymptomatic genetic carriers of HCM. These data support the hypothesis that mutation-related energy deficiency arises early in disease evolution. Mitochondrial dysfunction in HCM is tightly coupled with impaired organelle quality control and disrupted ultrastructure, marked by disorganized, low-density cristae and defective mitochondrial clearance. These alterations collectively amplify oxidative stress, disrupt myocardial bioenergetics, and exacerbate mitochondrial dysfunction, creating a vicious cycle that drives progression of the HCM phenotype [22].

### 2.2. ROS-Mediated Pathways in Hypertrophy Development

Calcium mishandling is a common downstream consequence of sarcomere mutations in HCM, and experimental studies show that a calcium-channel blockade partially prevents adverse remodeling in both MYH7 and TNNT2 mutant mice [23]. Ca^2+^/calmodulin protein kinase II (CaMKII) activation is associated with the transcription of pro-hypertrophic genes and was proposed as the main link between HCM-causal mutations and abnormal calcium signaling. However, in surgical samples from patients with HCM, Helms et al. observed that the post-translational activation of CaMKII was specific to sarcomere-mutation-related HCM. Conversely, phosphorylated histone deacetylase 4 (HDAC4), a CaMKII-dependent inducer of pro-hypertrophic transcription, shows comparable levels across HCM groups, underscoring the relevance of this pathway in sustaining hypertrophic remodeling [24]. Interestingly, ROS have been shown to promote the nuclear export of HDAC4 in pressure-overload hypertrophy [25], which supports the hypothesis of their potential role as key amplifiers of Ca^2+^ dysregulation and pathological hypertrophy in HCM. ROS can also activate extracellular signal-regulated kinases (ERKs) 1 and 2, which in turn modulate calcium homeostasis and hypertrophic gene expression [26]. In transgenic MYH7 R453C mice and MYH6 R453C piglets, Wang et al. observed that the over production of ROS was associated with the activation of both ERK1/2 and nuclear factor-κB (NF-κB) signaling pathways and the development of significant hypertrophy [27]. ROS-promoted lipid peroxidation and the accumulation of 4-hydroxy-2-nonenal (4-HNE) has been reported in HCM patients [28]. 4-HNE exerts an inhibitory action on AMPK, a metabolic sensor that suppresses anabolic growth pathways, thereby facilitating hypertrophic remodeling [29].

### 2.3. ROS-Induced Alteration in the Protein Quality Control System

The chronic imbalance between ROS generation and anti-oxidant pathways dysregulates the physiological protein quality control system (PQS), promoting myocardial fibrosis, stiffness, and apoptosis by several pathways [4,30].

On the one hand, post-translational modifications of titin, such as oxidation and ubiquitination, can decrease titin elasticity and impair the ubiquitin–proteasome system in HCM, thus modulating myocardial stiffness and contributing to the HCM phenotype [30]. On the other hand, the upregulation and S-glutathionylation of heat shock proteins (HSPs) such as HSP27 and α-ß crystalline has been associated with decreased myocardial stiffness in HCM, but at the cost of reduced cytoprotective effects [4].

### 2.4. Other Pathways Associated with a Disturbed Redox State in HCM

**Figure 1:**
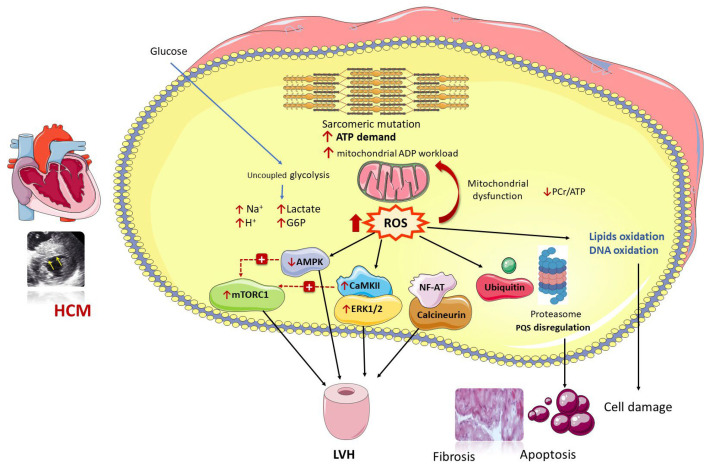
Oxidative–metabolic amplification loop in hypertrophic cardiomyopathy. Sarcomeric mutations alter sarcomere function and increase energetic cost and mitochondrial activity, which leads to mitochondrial dysfunction such as uncoupled glycolysis and impaired fatty acid oxidation. These alterations impact myocyte energetic efficiency by reducing the PCr/ATP ratio and affect the cardiac redox balance with increased oxidative stress. Excessive levels of cardiac ROS lead to LV hypertrophy via the activation of the AMPK, CAMKII/ERK 1/2, NFAT/calcineurin and mTORC1 pathways. The dysregulation of the PQS contributes to the development of fibrosis and alteration in the apoptotic mechanisms. The ROS-induced lipids and DNA oxidation contribute to the development of cell damage. ATP, adenosine triphosphate; CaMKII, calcium–calmodulin tyrosine kinase type II; ERK, extracellular signal-regulated kinase; G6P, glucose-6-phosphate; LVH, left ventricular hypertrophy; mTOR, mammalian target of rapamycin; NFAT, nuclear factor of activated T cells; PQS, protein quality control system; ROS, radical oxygen species.

In cardiomyocytes obtained from patients with advanced HCM undergoing cardiac transplant, Hassoun et al. observed a relationship between the high level of H_2_O_2_ and the concentration of pro-inflammatory cytokines such as intercellular cell adhesion molecule-1 (ICAM-1), vascular cell adhesion molecule-1 (VCAM-1), interleukin-6 (IL-6), interleukin-18 (IL-18), Toll-like receptor-2 (TLR2), and Toll-like receptor-4 (TLR4) [4]. A pro-inflammatory state was also associated with the perturbation of metabolic checkpoints, including MAPK, protein kinase B (Akt), mammalian target of rapamycin (mTOR), the forkhead box O transcription factor (FOXO), and c-Jun N-terminal protein kinase (JNK) [18]. Although no study has directly examined the interaction between ROS and mTOR specifically in HCM, several reports provide a mechanistic framework showing how ROS-activated kinases (CaMKII, Akt) and ROS-mediated AMPK suppression converge on mammalian target of rapamycin receptor-1 (mTORC1) activation, thereby promoting protein synthesis and hypertrophic remodeling [31]. Interestingly, mTOR inhibition ameliorated myosin heavy chain hypertrophy in a zebrafish model of HCM [32], whereas rapamycin, a well-known modulator of the mTOR pathway, was shown to prevent progressive LV hypertrophy in cats with subclinical HCM [33]. The main oxidative-metabomic amplification loop described in HCM are summarized in Figure 1.

## 3. Relationship Between Microvascular Dysfunction and Redox Imbalance in HCM

Microvascular dysfunction and blunted coronary response to adenosine are common features in HCM. In mouse models of HCM harboring MYBPC3 or MYH6 mutations, reduced myocardial capillary growth preceded the development of LV hypertrophy, suggesting that vascular alterations might occur early in the disease process [34]. The pathophysiological bases of microvascular dysfunction include extravascular compression due to HCM-related hypertrophy, diastolic dysfunction, and anatomic mutations of the small intramural coronary arterioles [34]. These alterations, together with cardiac fibrosis, myocyte disarray and reduced arteriolar density observed in HCM, predispose HCM patients to myocardial ischemia and contribute to the reduced energy supply to the heart and ROS production [35]. Endothelial dysfunction is also present in HCM, as shown by the blunted coronary flow in response to adenosine [36] and by the alteration in peripheral flow-mediated dilatation [37]. The precise mechanisms of these vascular alterations have not been clearly established. On the one hand, LV diastolic dysfunction and intrinsic alterations in the coronary structure might induce the microvascular flow-related changes observed in HCM. On the other hand, HCM is associated with the downregulation of genes associated with endothelial homeostasis [38], and with increased circulating biomarkers of endothelial dysfunction [39]. Even though the relationship between myocyte ROS and endothelial dysfunction has not been well addressed in HCM, observations in different clinical scenarios show that cardiomyocyte-derived ROS propagate to endothelial cells via ROS-induced ROS formation, activating endothelial nicotinamide adenine dinucleotide phosphate (NADPH) oxidases and mitochondrial ROS production. This redox transfer drives endothelial nitric oxide synthase (eNOS) uncoupling, and triggers nuclear factor-κB (NF-κB)-dependent inflammatory signaling, culminating in endothelial dysfunction and microvascular impairment [40]. Although the relative physiological importance of endothelium and myocyte-derived NO in the myocardial homeostasis remains to be established, Hassoun et al. observed a significant increase in activated monomeric eNOS and diminished NO bioavailability and soluble guanylate cyclase (sGC) activity in HCM [4]. The subsequent downregulation of the NO-sGC-3′,5′-cyclic guanosine monophosphate (cGMP)–protein kinase G (PKG) pathway may lead to hypophosphorylation of sarcomeric proteins, thereby increasing myocardial stiffness and contributing to cardiac hypertrophy through the suppression of the calcineurin–nuclear factor of activated T cells (NFAT) pathway [41].

## 4. Therapeutic Strategies in HCM

Current therapeutic strategies for hypertrophic cardiomyopathy remain limited and largely symptomatic, reflecting the absence of disease-modifying treatments for most patients. First-line management relies on negative inotropic agents -β-blockers, non-dihydropyridine calcium-channel blockers, and disopyramide—to reduce LV outflow tract obstruction and improve exertional symptoms—yet these agents do not halt disease progression. In obstructive forms refractory to medical therapy, septal reduction therapies such as surgical myectomy or alcohol septal ablation provide effective gradient relief but are invasive and available only in specialized centers [42]. The recent introduction of cardiac myosin inhibitors, including mavacamten, represents a significant advance, though their use is restricted by narrow therapeutic windows, the need for intensive echocardiographic monitoring, and uncertain long-term safety [43]. Importantly, no approved therapy directly targets the molecular drivers of hypertrophy, fibrosis, or microvascular dysfunction, leaving a substantial unmet need for disease-modifying interventions in HCM [44].

## 5. Oxidative Stress as a Therapeutic Target in Hypertrophic Cardiomyopathy

Because of the pivotal importance of oxidative stress in the pathophysiology of HCM, therapeutic strategies aimed at reducing the downstream pathways of ROS activation and restoring PQS—such as HSP induction [4], SGLT2i [45], NO signaling modulation [46], and mTOR inhibition [32,33]—may represent promising approaches to limit disease progression in HF and cardiomyopathies. Despite this, the experimental therapeutic evidences coming from HCM-specific models are scarce. N-acetylcysteine has been shown to reduce myocardial oxidative stress, stress-responsive signaling kinases, and fibrosis in a mouse model of HCM [47]. However, the HALT-HCM (Hypertrophy Regression With N-Acetylcysteine in Hypertrophic Cardiomyopathy) study—a single center, randomized controlled study—failed to prove the significance of the efficacy of N-acetylcysteine on indices of left ventricular hypertrophy and fibrosis [48]. Additional strategies, including HSP induction, aim to restore protein quality control systems through the stabilization of misfolded proteins and the reduction in proteotoxic stress [5]. The in vitro administration of HSPs (HSP27, α-ß crystallin and HSP70) to human HCM cardiomyocytes has been shown to diminish oxidative stress and cardiomyocyte stiffness [4]. mTORC1 inhibition has shown to be associated with reduced hypertrophy in experimental models of HCM [32,33]. Interestingly, anecdotal case reports have shown that rapamycin treatment is able to delay hypertrophy progression in infants with severe forms of the Noonan syndrome, a well-known HCM phenocopy [49,50].

## 6. Mechanisms of Action and Side Effects of SGLT2 Inhibitors

Sodium–glucose cotransporter 2 inhibitors (empagliflozin, canagliflozin, dapagliflozin and ertugliflozin) are antihyperglycemic drugs acting on sodium–glucose cotransporter 2 (SGLT2) expressed in the proximal convoluted tubules. These agents reduce the reabsorption of filtered glucose, decrease the renal threshold for glucose, and promote urinary glucose excretion, lowering HbA1c by about 0.7% [51]. Moreover, SGLT2i provoke an increase in distal tubular sodium load, resulting in the inhibition of the renin–angiotensin–aldosterone system [52]. Besides their impact on the improvement in glycemic control in type 2 diabetes mellitus [53], these molecules have been shown to significantly reduce major adverse cardiovascular events in patients with established cardiovascular disease [54] and improve the outcomes of patients with HF with reduced [55] and preserved left ventricular ejection fraction [6,44,56]. The pleiotropic effect of SGLT2i is due to their impact on crucial cellular processes such as nutrient signaling and anti-inflammatory pathways, as well as by promoting normal mitochondrial function, strengthening anti-oxidant defenses, and enhancing endothelial integrity [57]. At the endothelial level, increased SGLT2 mRNA and protein levels together with increased markers of oxidative stress and inflammation were observed in asymptomatic atherosclerotic plaques of diabetic patients undergoing carotid endarterectomy compared to non-diabetic patients, and to current users of SGLT2i [58]. Mroueh et al. demonstrated that SGLT2 protein levels had a positive correlation with oxidative stress and endothelial cell activation, and a negative correlation with eNOS levels [59]. Interestingly, inflammatory mediators such as the activated angiotensin II receptor type 1 (AT1R)/NADPH oxidases pro-oxidant pathway and angiotensin II, a key regulator of blood pressure and fluid balance, have been shown to mediate SGLT1 and SGLT2 expression in endothelial cells [60]. Conversely, the inhibition of SGLT2 has demonstrated anti-oxidant and endothelial protective effects, by decreasing ROS levels, enhancing mitochondrial function, and reducing the expression of pro-inflammatory cytokines such as tumor necrosis factor (TNF)-α, IL-6, and monocyte chemotactic proteins [61]. SGLT2 inhibitors are generally well tolerated, but their mechanism of inducing glucosuria and osmotic diuresis can lead to characteristic adverse effects. The most frequent adverse events are genital mycotic infections, which occur because of increased urinary glucose excretion, followed by mild urinary tract infections and volume depletion, particularly in older individuals or those receiving concomitant diuretics. Rare but clinically significant complications include euglycemic diabetic ketoacidosis, Fournier’s gangrene, and bone fractures. Despite these events, the overall incidence of serious adverse outcomes remains low, and the substantial cardiovascular and renal benefits of SGLT2 inhibition outweigh the associated risks [49].

## 7. Cardiac SGLT2 Expression

SGLT2 is constitutionally expressed in the kidneys. SGLT2 is also found at much lower concentrations in the brain, liver thyroid and skeletal muscle. The presence of SGLT2 within cardiomyocytes is still debated [62]. Single-cell transcriptomic analyses and studies using cardiomyocytes derived from human induced pluripotent stem cells have reported detectable levels of SGLT2 mRNA [63]. Further evidence was provided by Marfella et al., who identified SGLT2 expression in cardiomyocytes obtained from explanted failing hearts as well as from endomyocardial biopsies of diabetic and non-diabetic individuals [64]. Their observations, supported by immunofluorescence, immunohistochemistry, and FISH, were strengthened by confocal microscopy, which localized the transporter within cardiomyocytes using troponin T and wheat germ agglutinin markers. In subsequent work, the same group showed upregulation of the SGLT2 gene and protein expression in patients with low-flow, low-gradient aortic stenosis, associating these changes with markers of fibrosis, inflammation, and oxidative stress [65]. Interestingly, Mroueh et al. identified SGLT2 in human left ventricle (LV) and internal thoracic aorta samples and demonstrated that low-grade inflammation could directly stimulate the expression of SGLT2 and induce impairment in eNOS-NO/ROS balance [66]. Collectively, these findings highlight that SGLT2 may be expressed in cardiomyocytes at variable levels, and potentially upregulated in inflammatory or pathological states, contributing to the transition from healthy to disease state, and to the development of cardiac dysfunction, remodeling, and hypertrophy.

## 8. Molecular Mechanisms of SGLT2 Inhibition in the Heart

Sodium–glucose cotransporter 2 inhibitors have emerged as potent agents for reducing HF events, not solely through renal effects but via pleiotropic cardioprotective actions. In HF, ATP production is impaired due to mitochondrial dysfunction. This leads to the accumulation of toxic glucose and lipid byproducts that trigger nutrient surplus signaling (e.g., mTOR activation) and suppress cytoprotective nutrient-deprivation pathways, ultimately promoting cellular stress and damage [57].

SGLT2i might reverse these patterns in several ways.

(i) They modulate nutrient transport by inhibiting glucose transporter type 1 (GLUT1). In HF, glucose metabolism is dysregulated due to a shift from GLUT4 to GLUT1 transport, leading to excessive glycolysis, the accumulation of glucose-6-phosphate, and adverse O-GlcNAcylation of proteins [67]. SGLT2 inhibitors counteract this by downregulating GLUT1, enhancing GLUT4 expression, and improving glucose oxidation [68].
(ii) They modulate Na^+^ homeostasis by inhibiting sodium–hydrogen exchanger 3 (NHE3) [69] on the cardiomyocyte plasma membrane. This effect results in decreased cytosolic Na^+^ and Ca^+^ concentrations and increased fatty acid oxidation and mitochondrial health, which facilitate the mitochondrial clearance of toxic byproducts and the reduction in ROS production and inflammation [70,71,72,73].
(iii) They stimulate nutrient-deprivation signaling, reversing mTOR activation and improving oxidative phosphorylation and ATP synthesis [74].
(iv) They enhance long-chain fatty acid (LCFA) oxidation by upregulating CD36, fatty-acid-binding protein type 4 (FABP4), carnitine palmitoyltransferase (CPT)-1, and peroxisome proliferator-activated receptor alpha (PPARα) signaling while decreasing mTOR activity and increasing AMPK and peroxisome proliferator-activated receptor-γ coactivator 1-alpha (PGC1α) levels [75]. This shift reduces the accumulation of cytotoxic lipids like ceramide and diacylglycerol [76]. They also reduce the intracellular accumulation of branched-chain amino acids by increasing their degradation, thereby downregulating mTOR and improving cell viability [77].
(v) They increase the delivery of alternative fuels (ketone bodies and potentially short-chain fatty acids), which may act as signaling molecules rather than energy substrates. Ketonaemia induced by these drugs reduces inflammation, even without contributing significantly to ATP production [78].

Collectively, these mechanisms culminate in reversal of the fetal metabolic phenotype in the failing heart, reduction in cellular stress, and improvement in myocardial energetics and survival. Table 1 lists the main experimental studies showing the molecular mechanisms of action of SGLT2i in the heart.

## 9. Potential Benefits of SGLT2 Inhibitors in Hypertrophic Cardiomyopathy

As shown in the previous paragraphs, SGLT2 inhibitors exert significant anti-oxidant effects in cardiovascular tissues, independent of their glycemic actions. Experimental studies demonstrate that SGLT2 inhibition reduces mitochondrial and cytosolic ROS generation by suppressing NADPH oxidase activity and improving mitochondrial efficiency, thereby limiting oxidative injury [61]. In endothelial cells, these agents restore NO bioavailability by preventing eNOS uncoupling and reducing ROS-mediated NO scavenging, leading to improved redox balance and vascular function [83]. In cardiomyocytes, SGLT2 inhibitors attenuate oxidative stress-induced damage by stabilizing mitochondrial membrane potential, decreasing lipid peroxidation, and downregulating redox-sensitive pro-hypertrophic pathways such as CaMKII and mTOR [61]. Collectively, these findings support a direct role for SGLT2 inhibitors in modulating cardiac redox homeostasis, with potential therapeutic relevance for oxidative-stress-driven cardiomyopathies, including HCM. Despite these premises, evidence supporting the efficacy of SGLT2i across the broad spectrum of cardiomyopathies is limited. From a clinical perspective, randomized controlled trials evaluating SGLT2 inhibitors in HF have primarily focused on patients with reduced or preserved ejection fraction, including some with primary dilated cardiomyopathy, rather than other cardiomyopathies such as HCM [41,42,43,52]. However, the potential benefits of SGLT2 inhibitors in HCM includes:

(i) Inhibition of the sodium–hydrogen exchanger on the myocyte membrane. Ex vivo studies on isolated myocardial cells showed that SGLT2i inhibited sodium–hydrogen exchanger 1 (NHE)-1 on the myocyte plasma membrane, thereby lowering intracellular sodium and calcium levels [32,53,54]. In patients with HF, the activation of NHE-1 in the plasma membrane and the potential SGLT2 upregulation induces intracellular sodium and calcium accumulation in cardiomyocytes [55] which, in turn, enhances mitochondrial dysfunction and oxidative stress via ROS generation and activates AMPK-dependent transforming growth factor (TGF)-β signaling in cardiac fibroblasts [56,57,58]. Because altered calcium pathways, mitochondrial impairment, ROS generation and fibrosis are implicated in the genesis and progression of HCM [59,60], the impact of SGLT2 inhibitions on intracellular sodium and calcium homeostasis might have potential benefits on the evolution of the disease [61].
(ii) Modulation of metabolism. SGLT2i modulate cardiac energy metabolism by stimulating adipose tissue lipolysis and pancreatic glucagon release and suppressing insulin. These modifications increase circulating ketone concentrations and facilitate a metabolic shift from glucose oxidation to ketone body utilization as the predominant myocardial fuel source [62,63,84], which might contribute to counteract the altered energetics observed in HCM. By performing phosphorus magnetic resonance spectroscopy at rest and during peak dobutamine stress in 72 symptomatic patients with chronic HF receiving 12-week empagliflozin treatment, Hundertmark et al. did not find any significant change in the cardiac phosphocreatine:ATP ratio (PCr/ATP) [64]. Despite these results seeming to question the impact of gliflozins on myocardial energetics, the EMPA vision trial was plagued by several limitations, including its monocentric nature, the small recruited population and the relatively short treatment duration. Moreover, empagliflozin treatment was not associated with a significant improvement in functional capacity by the cardiopulmonary exercise test and natriuretic peptide reduction observed in previous clinical trials [42,44,52,65], suggesting that further studies would be necessary to clarify the impact of SGLT2i on myocardial metabolism.
(iii) Modulation of oxidative stress. As seen previously, oxidative stress has a pivotal role in the progression of HCM. SGLT2i might positively impact mitochondrial function, thereby improving myocardial energetics and reducing ROS production. On the other hand, the systemic and hemodynamic effects of SGLT2 inhibitors include the reduction in epicardial-adipose-tissue-related inflammation [66], the modifications of apolipoprotein profiles, and the reduction in circulating advanced glycosylated end products (AGEs), which are well-known sources of oxidative stress in myocardial cells [67]. Also, activation of the nutrient-deprivation pathway promoted by SGLT2i, was found to activate the AMPK/sirtuin (SIRT)/PGC-1α pathway [66,68,69,70], improving mitochondrial dysfunction, thereby attenuating oxidative stress, endoplasmic reticulum (ER) stress, inflammation and apoptosis after myocardial infarction in diabetic hearts [71].
(iv) Reduction in left ventricular hypertrophy, fibrosis, and adverse remodeling in preclinical and clinical models of HF [9,72] (Table 1).

**Figure 2:**
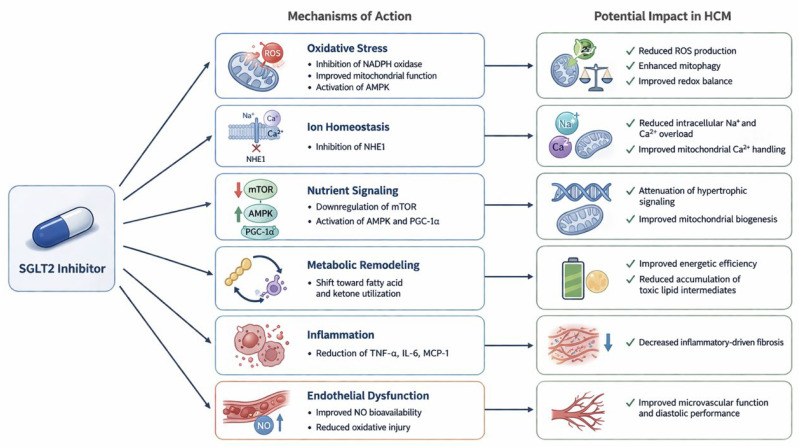
Integrated model of the proposed molecular mechanisms linking SGLT2 inhibition to therapeutic benefits in HCM. AMPK, adenosine monophosphate-activated protein kinase; IL-6, inteurleukin-6; MCP-1, monocyte chemoattractant protein-1; NO, nitric oxide; PGC-1α, peroxisome proliferator-activated receptor-γ, coactivator 1-α; ROS, reactive oxygen species; TNF-α, tumor necrosis factor-α. Icons included in this image are adapted from Servier Medical Art (https://smart.servier.com/), licensed under CC BY 4.0.

The main potential mechanisms of action of SGLT2i in HCM are summarized in Figure 2.

In the field of HCM, Wijnker et al. demonstrated that SGLT2i such as empagliflozin improved contractile relaxation in human engineered heart tissue derived from hiPSC-CMs with MYH7-R403Q mutations, a common HCM model. These effects were independent of SGLT2 receptor presence and, hence, may have been related to off-target effects via the activation of Na^+^/Ca^2+^ exchanger activity [20]. In mice with HCM induced by an R403Q mutation, Baka et al. showed that the administration of empagliflozin (15 mg/kg/day for 16 weeks) had a substantial impact on myocyte metabolism by reducing glucose uptake, uncoupled glycolysis and lactate production and increasing fatty acid oxidation. This effect was associated with a significant reduction in left ventricular hypertrophy and myocardial fibrosis as evidenced by echocardiography and histopathology [82]. In clinical practice, a retrospective analysis on HCM patients (*n* = 94) receiving SGLT2 inhibitors vs controls (*n* = 94) showed a positive impact of gliflozines on diastolic dysfunction, septal thickness, NTproBNP levels and NYHA functional class [85], with a satisfactory safety profile. Interestingly, in 2063 diabetic patients with HCM receiving SGLT2i, treatment was associated with a decreased risk of major adverse cardiac events [HR 0.76, 95% confidence interval (CI): 0.67–0.86], including a decreased risk of sudden cardiac death and ischemic stroke [86]. Finally in a large retrospective analysis of the TriNeXt Global Research Network, the outcome of 511 HCM patients on SGLT2i was compared with 28,150 HCM “control” subjects. After propensity score matching, treatment with SGLT2i was associated with lower rates of all-cause mortality (OR 0.24, *p* < 0.01), all-cause hospitalization (OR 0.69, *p* < 0.01) and cardiovascular symptoms (OR 0.63, *p* < 0.01) compared to patients not on SGLT2i [87]. Similar results were obtained in a large Mayo Clinic retrospective cohort, which showed that exposure to SGLT2i in HCM was associated with improvements in functional status and diastolic performance, lower incidence of HF hospitalization and a modest reduction in septal thickness [88]. The ongoing prospective SONATA-HCM (Sotagliflozin in Symptomatic Obstructive and Non-obstructive HCM) trial (NCT06481891) will allow confirmation of the safety and efficacy of SGLT2i in HCM.

## 10. Gaps in Knowledge and Perspectives

Despite compelling mechanistic and preclinical data in the potential therapeutic impacts of SGLT2i in HCM, several gaps in knowledge persist. First, until now only four retrospective studies have explored the benefit of SGLT2i in HCM, whereas most clinical evidence are derived from HF populations. Second, the presence and functional relevance of SGLT2 expression in cardiomyocytes remain debated [62,63,64]. Although some studies report detectable expression, cardioprotective effects are also observed in models lacking clear cardiac SGLT2 expression, suggesting possible off-target mechanisms [89]. Third, metabolic effects observed in HF populations may not uniformly translate to HCM, particularly given the differences in substrate utilization and the disease stage [20]. Finally, long-term safety and genotype-specific responses remain unexplored. These limitations highlight the need for dedicated mechanistic and clinical investigations in HCM populations.

## 11. Conclusions

Oxidative stress plays a central role in the pathophysiology of HCM linking genetic mutations to mitochondrial dysfunction, calcium mishandling, and maladaptive hypertrophy. SGLT2 inhibitors emerge as promising agents capable of restoring redox and energetic homeostasis through multifaceted mechanisms. While clinical validation in HCM populations is pending, existing molecular and preclinical data strongly support further investigation of SGLT2i as a disease-modifying therapy in hypertrophic cardiomyopathy.

## Figures and Tables

**Table 1:** Experimental studies underlying the cardiac benefits of SGLT2 inhibitors.

Study First Author (Ref.)	SGLT2 Inhibitor	Study Type	Experimental Setting	Molecular Target/Pathway	Key Findings
Wijnker et al. [20]	Canagliflozin, Dapagliflozin, Empagliflozin	Ex vivo	hiPSC-derived HCM (MYH7-R403Q)	Ca^2+^ handling/metabolism	Improved myocyte relaxation
Kolijn et al. [45]	Empagliflozin	Ex vivo	Human HFpEF myocardium	Oxidative stress/PKG signaling	↓ pro-inflammatory oxidative pathways, improved myocardial function
Ng et al. [63]	Empagliflozin	Ex vivo	hiPSC-cardiomyocytes	Glucotoxicity pathways	Reduction in hyperglycemia-induced cardiac dysfunction
Baartscheer et al. [79]	Empagliflozin	Ex vivo	Rat/rabbit cardiomyocytes	NHE1 inhibition (Na^+^/H^+^ exchanger)	↓ intracellular Na^+^ and improved ionic homeostasis
Koyani et al. [71]	Empagliflozin	In vitro and in vivo	Murine cardiomyocytes and adult mice	AMPK activation	AMPK-mediated protection against energy depletion
Wang et al. [72]	Empagliflozin	In vivo	Doxorubicin-induced cardiomyopathy in mice	Mitochondrial SIRT3/autophagy	Cardioprotection via the SIRT3 axis
Kondo et al. [73]	Canagliflozin	Translational	Human cardiomyocytes	Redox signaling	↓ oxidative stress and apoptosis via improvement in NOS coupling
Moellmann et al. [75]	Ertugliflozin	In vivo	C57BL/6J mice	mTOR/ER stress	↓mTOR signaling, ER stress, apoptosis
Aragón-Herrera et al. [76]	Empagliflozin	In vivo	Diabetic rats	Lipotoxicity (CD36, autophagy)	↓ cardiotoxic lipids, ↑ autophagy
Koizumi et al. [80]	Empagliflozin	In vivo	Diabetic rats	Mitochondrial ROS	↓ mitochondrial ROS and arrhythmogenicity
Yang et al. [81]	Empagliflozin	In vivo	Rats	AMPK/oxidative stress	↓ oxidative stress via AMPK activation
Baka et al. [82]	Empagliflozin	In vivo	HCM mouse (R403Q mutation)	Glucose vs FA metabolism	↓ glycolysis and lactate, ↑ FA oxidation → reduced hypertrophy and fibrosis

AMPK, AMP cyclic protein kinase; ER, endoplasmic reticulum; FA, fatty acid; HCM, hypertrophic cardiomyopathy; mTOR, mammalian target of rapamycin; ROS, reactive oxygen species; SIRT3, sirtuin 3.

## Data Availability

No new data were created or analyzed in this study.

## Abbreviations

The following abbreviations are used in the manuscript:

ADP	Adenosine diphosphate
AGEs	Advanced glycosylated end products
Akt	Protein kinase B
AMP	Adenosine monophosphate
AMPK	AMP-activated protein kinase
ATP	Adenosine triphosphate
AT1R	Calcium/calmodulin-dependent protein kinase II
cGMP	3′,5′-cyclic guanosine monophosphate
CPT1	Carnitine palmitoyltransferase 1
eNOS	Endothelial nitric oxide synthase
ERK 1	Extracellular signal-regulated kinase 1
ERK 2	Extracellular signal-regulated kinase 2
FABP4	Fatty Acid-Binding Protein type 4
FOXO	Forkhead Box O
GEO	Gene Expression Omnibus
GLUT1	Glucose transporter type 1
GLUT4	Glucose transporter type 4
HCM	Hypertrophic cardiomyopathy
HDAC4	Histone deacetylase 4
HF	Heart failure
4-HNE	4-Hydroxy-2-nonenal
H_2_O_2_	Hydrogen peroxide
HSP	Heart shock protein
JNK	Jun N-terminal Kinase
ICAM-1	Intercellular adhesion molecules-1
IL-6	Interleukin-6
IL-18	Interleukin-18
LCFA	Mitogen-activated protein kinase
MYBPC3	Myosin-binding protein
MYL3	Myosin light chain 3
MYH7	Beta-myosin heavy chain
mTOR	Mammalian target of rapamycin
mTORC-1	Mammalian target of rapamycin complex 1
NADH	Nicotinamide adenine dinucleotide
NADPH	Nicotinamide adenine dinucleotide phosphate
NADPH oxidase	Nicotinamide adenine dinucleotide phosphate oxidase
NFAT	Nuclear factor of activated T cells
NF-κB	Nuclear factor-κB
NHE3	Sodium/Hydrogen Exchanger 3
NO	Nitric oxide
O_2_•-	Superoxide anion radical
OH•	Hydroxyl radical
PCr	Phosphocreatine
PGC1α	Peroxisome Proliferator-activated Receptor-γ Coactivator 1-alpha
PKC	Protein kinase G
PPARα	Peroxisome proliferator-activated receptor alpha
PQS	Protein quality control
ROS	Reactive oxygen species
SIRT	Sirtuin
sGC	Soluble guanylyl cyclase
SGLT1	Sodium-glucose transporter 1
SGLT2	Sodium–Glucose transporter 2
SGLT2i	Sodium–Glucose transporter 2 inhibitors
TLR-2	Toll-like receptor-2
TNF-α	Tumor necrosis factor-α
TNNI3	Cardiac troponin I
TNNT2	Cardiac troponin T
TPM1	Tropomyosin alpha-1 chain
VCAM-1	Vascular cell adhesion molecule-1
